# Proteomics of Muscle Microdialysates Identifies Potential Circulating Biomarkers in Facioscapulohumeral Muscular Dystrophy

**DOI:** 10.3390/ijms22010290

**Published:** 2020-12-30

**Authors:** Victor Corasolla Carregari, Mauro Monforte, Giuseppe Di Maio, Luisa Pieroni, Andrea Urbani, Enzo Ricci, Giorgio Tasca

**Affiliations:** 1 Istituto di Biochimica e Biochimica Clinica, Università Cattolica del Sacro Cuore, 00168 Roma, Italy; corasolla@gmail.com (V.C.C.); giudimaio@hotmail.it (G.D.M.); andrea.urbani@policlinicogemelli.it (A.U.); 2 Unità Operativa Complessa di Neurologia, Fondazione Policlinico Universitario A. Gemelli IRCCS, 00168 Roma, Italy; mauro.monforte@gmail.com; 3 Unità di Proteomica e Metabolomica, Fondazione S. Lucia IRCCS, 00179 Roma, Italy; l.pieroni@hsantalucia.it; 4 Istituto di Neurologia, Università Cattolica del Sacro Cuore, 00168 Roma, Italy

**Keywords:** facioscapulohumeral muscular dystrophy, FSHD, inflammatory response, proteomics, biomarkers

## Abstract

Facioscapulohumeral muscular dystrophy (FSHD) is caused by a complex epigenetic mechanism finally leading to the misexpression of *DUX4* in skeletal muscle. Detecting DUX4 and quantifying disease progression in FSHD is extremely challenging, thus increasing the need for surrogate biomarkers. We applied a shotgun proteomic approach with two different setups to analyze the protein repertoire of interstitial fluids obtained from 20 muscles in different disease stages classified by magnetic resonance imaging (MRI) and serum samples from 10 FSHD patients. A total of 1156 proteins were identified in the microdialysates by data independent acquisition, 130 of which only found in muscles in active disease stage. Proteomic profiles were able to distinguish FSHD patients from controls. Two innate immunity mediators (S100-A8 and A9) and Dermcidin were upregulated in muscles with active disease and selectively present in the sera of FSHD patients. Structural muscle and plasminogen pathway proteins were downregulated. Together with the upstream inhibition of myogenic factors, this suggests defective muscle regeneration and increased fibrosis in early/active FSHD. Our MRI targeted exploratory approach confirmed that inflammatory response has a prominent role, together with impaired muscle regeneration, before clear muscle wasting occurs. We also identified three proteins as tissue and possibly circulating biomarkers in FSHD.

## 1. Introduction

Facioscapulohumeral muscular dystrophy (FSHD) is one of the most common muscular dystrophies and is characterized by initial weakness of the facial, shoulder and upper arm muscles although potentially progressing to affect almost all skeletal muscles [1].

In its most prevalent form, it is associated with the deletion of a subset of D4Z4 microsatellite repeats in the subtelomeric region of chromosome 4q which allows, in the presence of a polyadenylation signal sequence, the stable expression of the *DUX4* (double homeobox 4) retrogene. However, DUX4 protein and transcript levels cannot be easily measured in muscle tissue from FSHD patients [2], thus making the use of DUX4 as a biomarker unviable. The low level of its expression is in any case sufficient to determine several downstream molecular events, most notably including the activation of genes involved in germline and early stem cell development, innate immunity, and cancer testis antigens [3]. However, studies investigating the protein levels of these downstream targets in skeletal muscle and serum of FSHD patients are currently lacking.

Since FSHD presents a chronic, slow and highly variable clinical progression, proving the efficacy of a drug in a clinical trial is challenging [4]. Circulating biomarkers that reflect the degree of pathology could provide a quick, objective, and quantitative assessment of disease severity, allowing meaningful changes to be detected in a time period when functional changes may not yet be appreciated. Since all the muscles are exposed to the circulation, muscle derived serum proteins should theoretically reflect an average disease burden throughout the body.

To date, limited data are available on peripheral blood biomarkers in FSHD. Two pilot studies using commercially available multiplex assays [5] and high-throughput proteomic platforms [6] have identified an overrepresentation of a small number of proteins mostly related to unspecific muscle damage in FSHD sera compared with controls. While this manuscript was under revision, another study characterized plasma samples of individuals affected by early-onset FSHD using different -omics techniques [7], detecting a panel of miRNAs and the protein S100-A8 as candidate disease biomarkers.

Using muscle magnetic resonance imaging (MRI) to investigate FSHD, areas characterized by increased signal on short tau inversion recovery (STIR) sequences, accounting for muscle edema, were found in muscles not yet replaced by fat [8]. Following patients up over time, it is possible to appreciate an evolution towards fatty replacement of STIR hyperintense (STIR+) lesions [9]. STIR+ lesions are therefore considered a potential marker of disease activity in FSHD [10].

**Figure 1:**
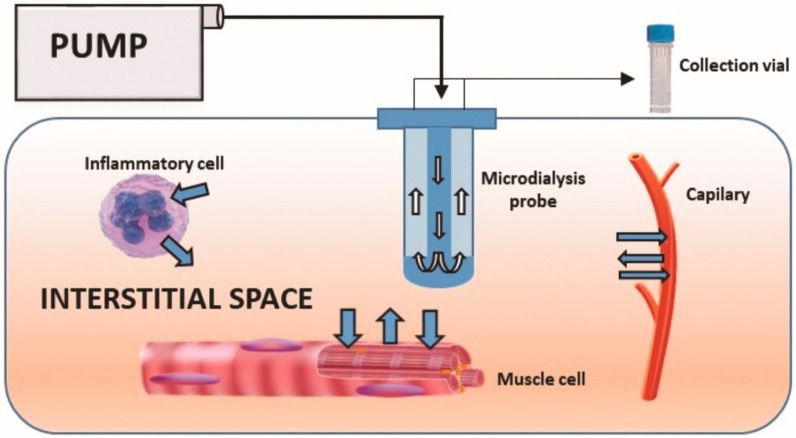
Schematic representation of the microdialysis system.

We previously conducted a microdialysis study on FSHD patients to identify the inflammatory cytokines present in the skeletal muscle intercellular milieu, using a multiplex assay analysis [11]. Microdialysis is a mini-invasive molecular sampling procedure performed with the introduction of a cylindrical semi-permeable membrane into the medium of interest, allowing a continuous molecular sampling of the interstitial fluid (Figure 1).

**Figure 2:**
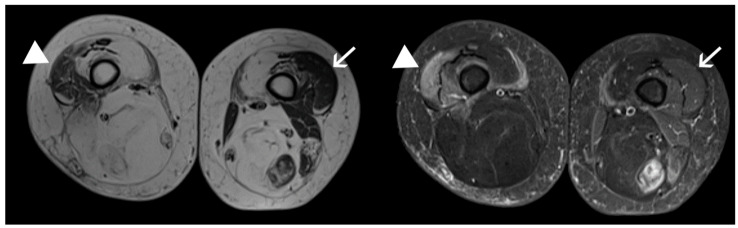
Muscles sampled by microdialyisis. STIR+ (arrowhead) and STIR- (arrow) vastus lateralis muscles sampled in the same patient. Axial STIR sequences of the thigh muscles are shown on the right-hand side, with corresponding T1-weighted images on the left.

We implemented a protocol using high cut-off membranes (100 kDa) targeting muscles with different MRI features (STIR+ and contralateral STIR-), simultaneously in the same patients (Figure 2). A summary of the characteristics of the population studied is reported in Table 1. 

In this work we used muscle interstitial fluid samples collected by microdialysis to look more broadly at the composition of the extracellular milieu. We applied a holistic approach, by means of shotgun proteomics, to characterize and compare the whole protein set present in the different samples, followed by a targeted approach trying to identify known DUX4 targets at the protein level. Finally, we analyzed the protein signature of serum samples from the same patients.

## 2. Results

### 2.1. Combined Proteomic Analysis of Microdialysates

**Figure 3:**
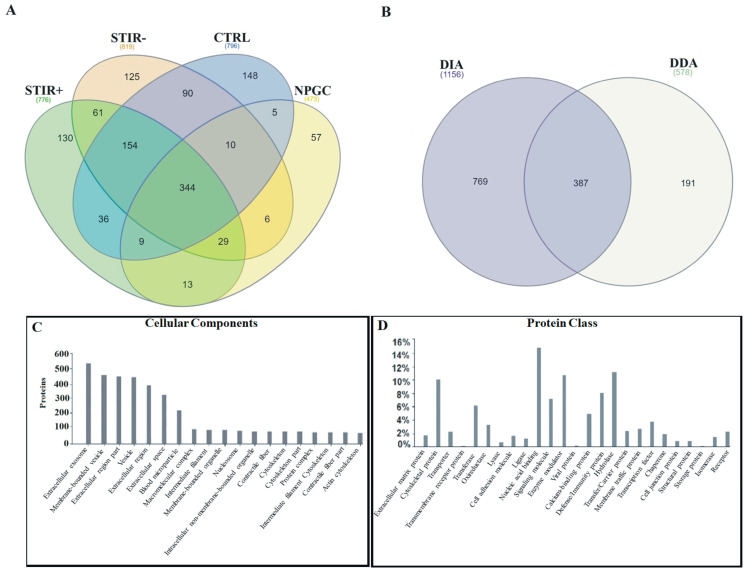
Identified proteins and Gene Ontology analysis. (**A**) Venn diagram of all the proteins identified in DIA in the examined conditions. (**B**) Overlap between proteins identified with the Synapt G2Si (DIA) and Orbitrap Elite (DDA) mass spectrometers. (**C**) PANTHER Gene Ontology analysis showing the cellular components to which the identified proteins belong. (**D**) PANTHER Gene Ontology analysis showing the class distribution of the identified proteins.

We identified by Data Independent Acquisition (DIA) a total of 1156 proteins of which 344 were shared among all the conditions, 130 were found only in the samples from the STIR+ muscles, 125 only in STIR-, 148 in controls and 57 in the non-penetrant gene carrier (NPGC) (Figure 3A; Supplementary Table S1). To increase the robustness of data, microdialysate samples from 3 STIR+, 3 STIR-, and 3 control muscles were submitted to a different setup of analysis in Data Dependent Acquisition (DDA) mode. A total of 578 proteins were identified with high confidence (Supplementary Table S2). Comparing the identified proteins obtained from both of mass spectrometers we observed a broad overlap, since 67% of the proteins identified by DDA were also covered by DIA (Figure 3B).

A functional classification analysis using PANTHER was performed on the whole list of identified proteins. The classification according to the Gene Ontology (GO) term Cellular Component showed that the proteins recovered in the microdialysates mostly belonged to the extracellular space component confirming their secretory origin, in accordance with the provenience of the samples (Figure 3C). Nucleic acid binding (16%), enzyme modulator (11.6%) and defense immunity (9.6%) were found to be among the most populated subgroups of the functional classification according to the GO term Protein Class (Figure 3D).

### 2.2. Comparative Proteomic Analysis Using DIA: STIR+ Versus STIR- Muscles

Using a label free method, we quantified and identified dysregulated proteins in STIR+ compared with STIR- muscles. We found 21 proteins upregulated in STIR+ muscles (fold change higher than 20%). Among those, Prostaglandin-H2 D-isomerase (P41222), Protein S100-A9 (P06702), Insulin-like growth factor-binding protein 4 (P22692), Dermcidin (P81605), and several Immunoglobulin kappa fragments showed an upregulation higher than 50%.

On the contrary, 29 proteins, mainly represented by structural muscle proteins, were found downregulated in STIR+ muscles (Table 2).

**Figure 4:**
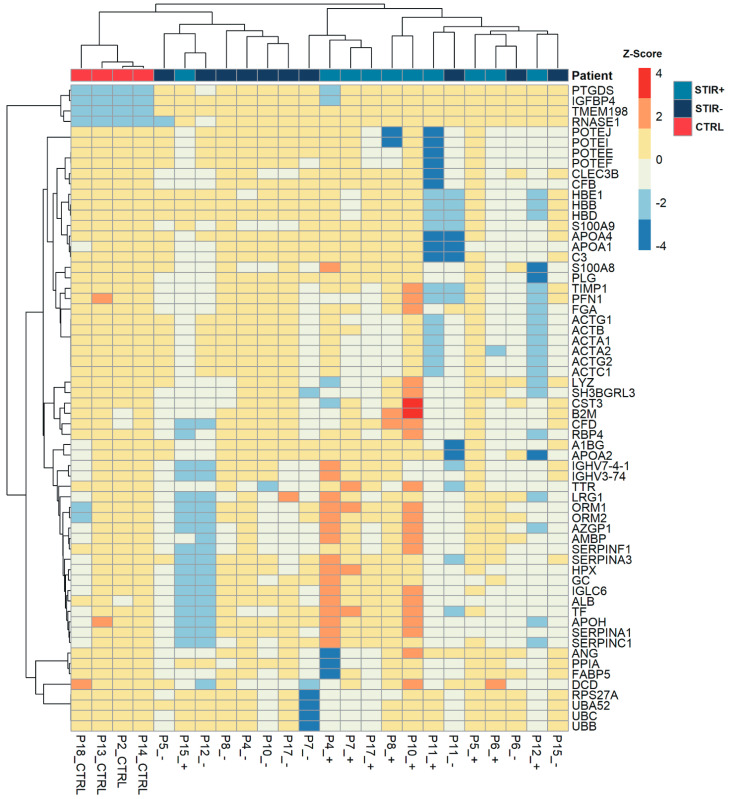
Heatmap showing the expression level of the proteins in STIR+, STIR- and control samples.

The heatmap of all the STIR+, STIR- and control samples showed that controls were gathered together and displayed clearly higher similarity to STIR- than STIR+ samples. Nine out of 10 STIR+ samples also clustered in the same group. Three STIR- samples (P6, P11 and, to a lesser extent, P7) clustered close to their correspondent STIR+ (Figure 4). 

The paired analysis of samples obtained from the STIR+ and contralateral STIR- muscle of the same patient confirmed a significant dysregulation of eight proteins (Supplementary Figure S1). Other proteins of possible interest, such as components of complement system (Complement C1q subcomponent subunit B (P02746); Complement C1r subcomponent (P00736); Complement C1s subcomponent (P09871); Complement component C8 gamma (P07360) and beta (P07358) chains) [12] were identified only in a in small number of STIR+ samples; several C-C motif chemokines (14, 18, 21, and 27) were also upregulated in the STIR+ samples analyzed, even if not reaching statistical significance (Supplementary Table S3).

Ingenuity pathway analysis (IPA) revealed that dysregulated proteins in STIR- versus STIR+ samples predict the activation in STIR- muscles of *MRTFA* (myocardin related transcription factor A), *MRTFB* (myocardin related transcription factor B), *SRF* (serum response factor), and *NFE2L2* (nuclear factor, erythroid 2 like 2), a master regulator of antioxidant response in injury and inflammation (Supplementary Table S4). On the contrary, *IFNG* (interferon gamma) is predicted to be inhibited in STIR- compared with STIR+ muscles. IPA also showed modulations in the inflammatory response, metabolic, and developmental pathways (Supplementary Table S5; Figure S2A).

### 2.3. Comparison between STIR+ Muscles and Controls

A total of 84 proteins were dysregulated of which 30 showed an upregulation in STIR+ muscles, while 54 were downregulated. The upregulated proteins were mainly involved in complement and coagulation cascades and innate immune response. The quantitative analysis confirmed a downregulation in STIR+ muscles of several structural muscle proteins, consistently with the signs of early damage seen on MRI, of proteins related with the plasminogen pathway (Plasminogen, Plasminogen-like protein B and Vitronectin), as well as of Neutrophil defensin 1 and 3 (Supplementary Table S6).

IPA showed that the protein regulation in STIR+ muscles could be affecting among others the clathrin-mediated endocytosis and LXR/RXR activation pathways (Supplementary Table S5). The upstream activation analysis suggested a possible regulation of the genes *SRF*, *NOS2* (nitric oxide synthase 2), *MYOD1* (myogenic differentiation 1), and *IL6* (interleukin 6) (Supplementary Table S4). Finally, network analysis supported an alteration of skeletal muscle system development and function and inflammatory response (Supplementary Figure S2B).

### 2.4. Comparison between STIR- Muscles and Controls

In total, 135 proteins were differentially regulated. Several of the proteins upregulated in STIR+ were also found upregulated in STIR- patients compared to controls (Supplementary Table S7). Insulin-like growth factor II (P01344) was found only in the STIR- muscles; Fatty acid binding protein_adipocyte (P15090) was six-fold more expressed in STIR- samples, while Complement factor D (P00746) and Flavin reductase (P30043) were three-fold more expressed. Structural muscle proteins were downregulated also in the STIR- samples, although to a lesser extent than in STIR+ samples.

Upstream analysis confirmed regulation of pathways overlapping with the STIR+ versus control comparison. We found a predicted activation of *KDM5A* (lysine demethylase 5A) and a predicted inhibition of *MYOCD* (myocardin), *IPMK* (inositol polyphosphate multikinase) and *MEF2C* (myocyte enhancer factor 2C), that are genes involved in myogenesis and muscle differentiation (Supplementary Table S4). IPA Network analysis confirmed the association of the dysregulated proteins in STIR- muscles with cellular assembly and organization, muscle development and function (Supplementary Figure S2C). When the NPGC sample was analyzed using the same parameters of quantitative analysis and compared to all the other conditions, it showed greater similarity with controls and only four unique proteins were identified (Supplementary Table S3).

### 2.5. DDA Label Free Quantification

Among the 578 proteins identified, 31 were differentially expressed in STIR+ compared to the STIR- and control samples, the majority being upregulated ≥30% (Table 3). Most of dysregulated proteins in both DDA and DIA, such as Beta-2-microglobulin (P61769), S100-A8 (P05109), A9 (P06702), Dermcidin (P81605), and Transthyretin (P02766), were significantly upregulated in STIR+ muscles. Structural muscle fiber proteins were consistently downregulated in samples from STIR+ muscles also in the DDA mode.

### 2.6. Targeted Proteomic Approach

We analyzed a pool of all the digested peptides from STIR+ and STIR- muscles, identifying 515 proteins in total, and performed multiple reaction monitoring (MRM) targeted analysis choosing a list of proteins related with DUX4 expression [3] to be specifically selected by the instrument. Despite this targeted approach, none of these proteins could be identified in the microdialysates.

### 2.7. Serum Analysis

**Figure 5:**
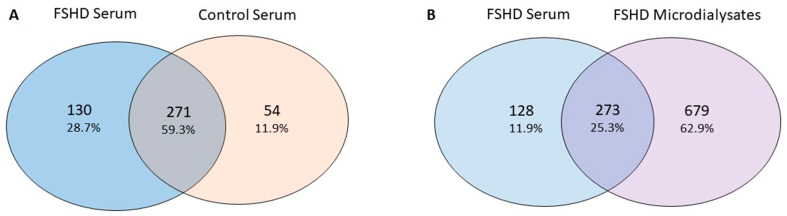
Overlaps of proteins identified in the serum. (**A**) Overlap between proteins identified in FSHD and healthy control sera after albumin and IgG/IgM removal. (**B**) Overlap between proteins identified in FSHD patients’ sera and microdialysates.

Approximately 70% of the albumin and IgG content was removed after column treatment. We were able to qualitatively identify 455 proteins in at least one subject of which 130 were found only in FSHD patients, 271 were in common between FSHD and controls and 54 were present only in controls (Figure 5A and Supplementary Table S8).

Expression analysis comparing FSHD serum samples against healthy controls showed 19 dysregulated proteins, CD5 antigen-like (O43866) being the most upregulated (1.6 fold-change) (Supplementary Table S9). The majority of the proteins found in the serum of FSHD patients were also present in the microdialysates (*n* = 273, Figure 5B). Among these 273, 52 were present in at least one of the STIR+ microdialysate samples and were absent in the serum of controls, thus configuring potential circulating biomarkers of disease activity in FSHD. Among these 52 proteins, Dermcidin, S100-A8, and A9 were found to be upregulated in the microdialysates of 8/10 STIR+ muscles (Supplementary Figure S3).

## 3. Discussion

Biomarker identification is crucial both in the perspective of forthcoming clinical trials in FSHD and also to shed more light on the still elusive pathomechanisms taking place in vivo in this disease. Circulating biomarkers can represent an ideal index to monitor overall disease progression, in accordance with the concept of “liquid biopsies”. Liquid chromatography-mass spectrometry (LC-MS) has been largely used for biomarker investigation, thanks to its capacity to identify and quantify large numbers of proteins with great accuracy. Many studies using proteomic approaches identified possible biomarkers for cancer, neurodegenerative diseases as well as muscular dystrophies [13]. In this context, this is the first study to examine microdialysates from patients’ muscles stratified by MRI using a bottom-up proteomic approach. We found that most of the proteins in peripheral blood were also present in the microdialysates, confirming their secretory origin from skeletal muscle, which represents the most abundant tissue of our body, and in accordance with a recent proteomic profiling of DUX4 overexpressing cells that showed an upregulation of pathways related to exocytosis [14].

Compared with capture-based strategies, LC-MS has the advantage of truly hypothesis-free biomarker discovery [15]. On the other hand, a potential disadvantage is that peptides from highly abundant proteins can mask or interfere with peptides from less abundant proteins, thus decreasing the sensitivity of the assay [16]. However, the significant number of identified peptides in our study demonstrates that LC-MS can outperform multiplex targeted techniques [6].

Aiming to further increase the reliability of the data we chose to validate our results in two different mass spectrometry (MS) platforms. This strategy was used since the DDA mode provides a selective, quantitative and sensitive analysis by selecting peaks with higher intensity in each MS cycle and submitting them to a subsequent fragmentation. The principal advantage of this method is represented by the high accuracy for selected parent ions at the expense of a lack of acquisition of proteins excluded from this transition list. Orbitrap technology has the additional advantage of being commonly used also because of an easier MS/MS spectra interpretation [17]. The overlap between DDA and DIA approaches using two different MS platforms argues in favor of a good accuracy and reproducibility of the results.

Consistently with the presence of early damage in STIR+ muscles, most of the structural muscle proteins were downregulated in these samples. Other proteins found modulated in STIR+ muscles, such as Carbonic anhydrase 1 and 2, Neutrophil Defensin 1 and 3, and proteins involved in complement system/innate immune response activation (Table 2), have been already described as dysregulated and possibly linked with FSHD pathophysiology [6,11].

We also report that S100-A8, S100-A9, Dermcidin and Insulin-growth factor-binding 4 were significantly (more than 50%) upregulated in STIR+ muscles compared with STIR-, together with Prostaglandin-H2 D-isomerase. The latter catalyzes the conversion of Prostaglandin-H2 to Prostaglandin-D2, a prostaglandin that has been found in Duchenne muscular dystrophy patients associated with muscle necrosis; assessment of its metabolites in urines has been proposed as a biomarker of progression in that disease [18]. Interestingly, S100-A8 and A9 and Dermcidin were found consistently upregulated in the samples of STIR+ muscles both by DIA and DDA. S100-A8 and A9 are calcium binding proteins actively secreted by myeloid cells (both neutrophils and monocytes), supposed to play a pivotal role in the pathogenesis of rheumatoid arthritis, inflammatory bowel disease and cystic fibrosis [19,20]. They constitute a heterodimer, also known as calprotectin, which may act as a damage-associated molecular pattern (DAMP) [21] able to activate innate immune pathways through stimulation of Toll-like receptor 4, therefore constituting a key molecule to trigger stress and inflammatory responses also in cancer [22]. S100-A8 and A9 have been reported as potential biomarkers in systemic autoimmune diseases [23], and given their function in the amplification of the inflammatory cascade could also constitute a possible therapeutic target. Notably, S100-A8 and Prostaglandin-H2 D-isomerase have been recently found significantly upregulated in the blood of early-onset FSHD patients compared with controls by LC-MS. For S100-A8, the finding was also confirmed by enzyme-linked immunosorbent assay [7].

Dermcidin is instead secreted by sweat glands on the skin, where it exerts antimicrobial activity. However, it is also secreted by skeletal muscle and the full-length protein promotes apoptosis under hypoxic conditions [24], while proteolytic fragments have the opposite role of promoting cancer cell survival in stress conditions [25]. Dermcidin has also recently been proposed as a prognostic biomarker of lung cancer together with S100-A9 [26].

Transcriptional upregulation of complement genes was one of the major findings in STIR+ muscles in two MRI targeted studies [12,27]. At protein level, complement system was among the top canonical dysregulated pathways in the extracellular fluid and we found consistently higher levels of complement factor D in both STIR+ and STIR– muscles compared to controls. Complement factor D activates C3b through the binding with factor B in the antibody independent, alternative complement pathway [28].

Plasminogen-like protein B, Plasminogen, and Vitronectin were found to be downregulated in STIR+ muscles (Table 2 and Supplementary Table S6), consistently with an overall downregulation of the plasminogen pathway. Indeed, plasminogen system activation has a proteolytic role in the extracellular matrix which is instrumental for muscle regeneration [28,29]. A perturbation in the delicate balance of inflammatory response and extracellular matrix remodeling, that provides the scaffold for the formation of new myofibers, might contribute to an impairment in the regenerative capacity in FSHD and eventually to the accumulation of fibrotic tissue. Concordantly, myogenic factors like *MYOD1* and its target *MEF2C* are predicted to be inhibited in FSHD muscles, as previously reported in early microarray studies [30]. Vitronectin interacts directly and indirectly with plasminogen but also bridges it with complement, being one of the main inhibitors of the terminal pathway of the complement system [30,31]. Finally, Protein AMBP, that was found ~2 times more expressed in STIR+ samples when compared with control samples, can inhibit plasmin, which is the major enzyme responsible for the dissolution of fibrin as well as components of the extracellular matrix [32].

IPA highlighted an upstream activation of the MRTF/SRF axis in STIR- samples when compared to STIR+. MRTF-A and B interact with SRF and are involved in several cellular processes including skeletal muscle development and myogenic differentiation [33], and a downregulation of the MRTF/SRF signaling pathway is present in muscle atrophy secondary to disuse [34]. The (partial) shutdown of this pathway in STIR+ muscles could be also consistent with a deficiency of regenerative capacity in affected FSHD muscles [35]. IPA further confirmed a perturbation in humoral immune response, immune cell trafficking and tissue development in STIR+ muscles.

We also provide preliminary evidence of protein modulation in FSHD sera applying the same MS protocol used for the microdialysates. CD5 antigen-like was found in the samples of all the patients significantly upregulated compared with controls. It is a key regulator of intracellular lipid synthesis and, notably, is mainly expressed by macrophages in lymphoid and inflamed tissues where it regulates several processes including fibrosis [36]. It has also been proposed as a biomarker in a number of inflammatory [36,37] and neoplastic conditions [38].

Among the other proteins that were present in the serum of at least one patient and absent in controls, three (S100-A8, A9 and Dermcidin) could be identified in 80% of the analyzed microdialysates derived from STIR+ muscles. Given that these proteins were also consistently present among the most upregulated in STIR+ muscles with both types of proteomic analysis implemented, they configure the strongest candidates as circulating biomarkers of disease activity identified with our study.

Our study has some limitations. The first one is related to its exploratory nature on a small number of patients and to the very small amounts of interstitial fluid available [11], thus providing results that need to be confirmed on larger numbers and with different methods. The second one is constituted by the filter applied on the microdialysis membrane, whose cut-off could affect the recovery of potential biomarkers with higher molecular weight. The third is an intrinsic drawback of the proteomic approach when dealing with sera, where target proteins are diluted and suffer from a possible ion suppression by the most abundant proteins.

## 4. Materials and Methods

### 4.1. Patients and Samples

Patients were consecutively enrolled during the MRI follow-up in the period April 2014–February 2016, corresponding to the time window of our previous study [11]. Briefly, patients had to have one externally accessible lower limb muscle showing normal signal on T1-weighted MRI sequences and hyperintense signal on STIR sequences, i.e., one muscle showing edema changes but not yet replaced by fat tissue, together with the contralateral muscle showing normal signal on both T1-weighted and STIR sequences. FSHD patients underwent microdialysis on the identified STIR+ and STIR- muscles at the same time, while only one muscle was studied for controls and NPGC. Subjects with concomitant systemic inflammatory diseases or under treatment with anti-inflammatory drugs were excluded. We collected microdialysis and blood samples from 10 genetically confirmed typical FSHD patients (5 females and 5 males, mean age 41.7 ± 13.4, range 18–58), 4 controls (3 males and 1 female, mean age 41.5 ± 16.4 years, range 24–60) and one NPGC (female, age 47). Controls were healthy subjects who volunteered to undergo the microdialysis procedure, including unaffected relatives of FSHD patients, with age >18 and <65 years, and no systemic inflammatory disease. Clinical and genetic data of all subjects is reported in Table 4. The microdialysates selected for proteomic analysis were obtained at comparable time points between 48 and 56 h post catheter insertion.

This protocol is in agreement with the Declaration of Helsinki and was approved by the Ethics Committee of our Institution. All the involved subjects gave their written informed consent prior to the inclusion in the study.

### 4.2. Mass Spectrometry Based Proteomic Analysis

The label free shotgun proteomic analysis was performed using two different experimental setups: DIA performed on a Synapt G2-Si Q-TOF mass spectrometer with T Wave cell for ion mobility separation (Waters Corp.), and DDA, performed on a Orbitrap Elite mass spectrometer (Thermo) [39].

### 4.3. Synapt G2Si- Proteomic Analysis

Twenty-five microdialysis samples from 10 FSHD patients (simultaneously obtained from 10 STIR+ and 10 contralateral STIR- muscles), 4 controls and the NPGC were quantified using the Bio-Rad Protein Assay method and had an average protein concentration of approximately 0.5 μg/μL. To minimize the problem of the low volume and protein concentration, we applied the filter-aided sample preparation (FASP) protocol [40]. For the FASP protocol we made use of a microcolumn tip with 10kDa molecular weight cut-off (MWCO). Ten micrograms of protein amount were diluted into a final volume of 100μL in Tris-Urea Buffer (Tris-HCl 100mM pH 7.5; Urea 6M) and applied in the column for further reduction, alkylation and trypsin digestion.

Serum samples were first desalted in a spin filter column (Vivaspin 3000 Da MWCO): 250 μL of each sample were added into a spin filter and centrifuged for 60 min at 5000g, and the material retained in the filter was used to perform Albumin and IgG/IgM removal (Pierce Albumin/IgG removal Kit, Thermo Scientific) according to the manufacturer’s instructions. After the process of desalination and removal of Albumin and IgG, the leftover proteins were washed, and the two fractions were later submitted to the same digestion protocol already mentioned. 

The peptides obtained were submitted to a LC-MS analysis, using a High Definition Synapt G2-Si mass spectrometer directly coupled to the chromatographic system. For each sample the same amount of digested peptides was analyzed. Continuum LC-MS data from three replicate runs for each were processed for qualitative and quantitative analysis using the software ProteinLynx Global Server v. 3.0.3 (PLGS, Waters Corp.) [41].

### 4.4. Orbitrap Elite Proteomic Analysis

Protein concentration of 3 microdialysates for each condition (STIR+ and STIR- muscles from P7, P8, and P10, and controls P13, P14, and P18, selected based on the amount of microdialysate material left after previous analyses) was assayed by Bio-Rad Protein Assay and 7.6 µg were used for further processing. To reduce the impact of the most abundant proteins, a fractionation method was carried out [42].

Additional details about MS instrument settings, statistical analysis, label free quantification, MRM, network analysis, and heatmap building are available as Supplementary Methods.

## 5. Conclusions

Our pilot study proved that high-resolution proteomics is a feasible method to analyze muscle microdialysates, and that proteomic profiles of interstitial muscle fluids are able to distinguish FSHD patients from controls. The modulation of protein expression in FSHD muscles with signs of early damage showed the activation of pathways related to inflammation, which further supports the role of this process in active disease phases, and a lack of regenerative capacity. We propose a “protein signature” associated with active disease constituted by the downregulation of structural muscle proteins and upregulation of Dermcidin, S100-A8, and A9.

## Figures and Tables

**Table 1:** Summary of the characteristics of study participants. Age is reported as mean ± standard deviation; for EcoRI fragment length (in kb) and Clinical Severity Scale (CSS) the median value is indicated. M = males, F = females.

CATEGORY	*n*	AGE	SEX	EcoRI	CSS
FSHD	10	41.7 ± 13.4; range 18–58	5 M–5 F	21.5; range 15–25	3; range 1.5–3.5
CONTROLS	4	41.5 ± 16.4; range 24–60	3 M–1 F	---	---

**Table 2:** Comparison of protein level expression between STIR+ and STIR- samples, shown as ratio.

Accession Number	Protein Description	Score	STIR+:STIR- Ratio
P41222	Prostaglandin-H2 D-isomerase (PTGDS)	661.92	2.89
P06702	Protein S100-A9 (S100A9)	4710.25	2.23
P22692	Insulin-like growth factor-binding protein 4 (IGFBP4)	450.89	1.80
P81605	Dermcidin (DCD)	3227.52	1.77
A2NJV5	Immunoglobulin kappa variable 2-29 (IGKV2-29)	2955.26	1.67
A0A075B6S2	Immunoglobulin kappa variable 2D-29 (IGKV2D-29)	2955.26	1.65
A0A075B6P5	Immunoglobulin kappa variable 2-28 (IGKV2-28)	2955.26	1.65
P06310	Immunoglobulin kappa variable 2-30 (IGKV2-30)	2955.26	1.63
A0A0A0MRZ7	Immunoglobulin kappa variable 2D-26 (IGKV2D-26)	2955.26	1.63
A0A075B6S6	Immunoglobulin kappa variable 2D-30 (IGKV2D-30)	2955.26	1.62
P01615	Immunoglobulin kappa variable 2D-28 (IGKV2D-28)	2955.26	1.62
P01614	Immunoglobulin kappa variable 2D-40 (IGKV2D-40)	3081.8	1.62
A0A087WW87	Immunoglobulin kappa variable 2-40 (IGKV2-40)	3081.8	1.58
P05109	Protein S100-A8 (S100A8)	3410.17	1.42
P01619	Immunoglobulin kappa variable 3-20 (IGKV3-20)	11,910.68	1.42
P02766	Transthyretin (TTR)	21,752.78	1.34
P02790	Hemopexin (HPX)	10,037.44	1.31
P11217	Glycogen phosphorylase_ muscle form (PYGM)	669.66	1.26
P07998	Ribonuclease pancreatic (RNASE1)	3437.69	1.26
P11216	Glycogen phosphorylase_ brain (PYGB)	632.03	1.25
P02652	Apolipoprotein A-II (APOA2)	18,158.41	1.23
P59665	Neutrophil defensin 1 (DEFA1)	3886.69	0.81
P59666	Neutrophil defensin 3 (DEFA3)	3886.69	0.79
P62937	Peptidyl-prolyl cis-trans isomerase A (PPIA)	891.54	0.73
Q9H299	SH3 domain-binding glutamic acid-rich-like protein 3 (SH3BGRL3)	2135.99	0.73
P61626	Lysozyme C (LYZ)	6273.03	0.71
P02042	Hemoglobin subunit delta (HBD)	29,240.84	0.71
P07451	Carbonic anhydrase 3 (CA3)	1277.63	0.70
P0CG47	Polyubiquitin-B (UBB)	685.65	0.69
P62987	Ubiquitin-60S ribosomal protein L40 (UBA52)	685.65	0.68
P62979	Ubiquitin-40S ribosomal protein S27a (RPS27A)	690.87	0.67
P0CG48	Polyubiquitin-C (UBC)	685.65	0.67
P68871	Hemoglobin subunit beta (HBB)	67,024.84	0.65
P69905	Hemoglobin subunit alpha (HBA1)	28,016.07	0.65
P02671	Fibrinogen alpha chain (FGA)	3726.94	0.63
P06727	Apolipoprotein A-IV (APOA4)	3903.4	0.59
P00747	Plasminogen (PLG)	2244.15	0.57
P02100	Hemoglobin subunit epsilon (HBE1)	1198.27	0.48
P07737	Profilin-1 (PFN1)	8113.64	0.47
A5A3E0	POTE ankyrin domain family member F (POTEF)	354.23	0.43
Q02325	Plasminogen-like protein B (PLGLB1)	895.85	0.42
Q6S8J3	POTE ankyrin domain family member E (POTEE)	363.36	0.42
P69891	Hemoglobin subunit gamma-1 (HBG1)	2857.95	0.41
P63261	Actin_ cytoplasmic 2 (ACTG1)	2756.19	0.40
P60709	Actin_ cytoplasmic 1 (ACTB)	2756.19	0.40
P69892	Hemoglobin subunit gamma-2 (HBG2)	2888.5	0.39
P68032	Actin_ alpha cardiac muscle (ACTC1)	1401.05	0.33
P63267	Actin_ gamma-enteric smooth muscle (ACTG2)	1401.05	0.32
P62736	Actin_ aortic smooth muscle (ACTA2)	1401.05	0.32
P68133	Actin_ alpha skeletal muscle (ACTA1)	1401.05	0.32

Only proteins with more than 20% fold-change are listed. Score is calculated through the combined scores of all identified mass spectra that can be overlaid to amino acid sequences within each protein. Higher scores indicate a more confident match (ProteinLynx Global Server, PLGS, cutoff score: 4.5). Only proteins with *p*-value ≤ 0.05 are shown.

**Table 3:** Label free analysis of proteins identified by DDA.

Accession Number	Protein Description	PSMs STIR+	PSMs STIR-	PSMs CTRL	Fold Change STIR+/STIR-	Fold Change STIR+/CTRL	*p* Value STIR+/STIR-	*p* Value STIR+/CTRL
Q8NE71	ATP-binding cassette sub-family F member 1 (ABCF1)	3.5	1	1	3.5	3.5	0.007	0.007
P13796	Plastin-2 (LCP1)	4	1	12.5	4	−3.13		0.003
Q14019	Coactosin-like protein (COTL1)	4	1	6.5	4	−1.63		0.038
P61769	Beta-2-microglobulin (B2M)	27	14	11.5	1.93	2.35	0.012	0.029
P04040	Catalase (CAT)	35.5	16	8.5	2.22	4.18	0.040	0.017
P81605	Dermcidin (DCD)	19	8.5	13	2.24	1.46	0.020	
O75223	Gamma-glutamylcyclotransferase (GGCT)	9.5	3	3	3.17	3.17	0.006	0.006
P15924	Desmoplakin (DSP)	65.5	29.5	7.5	2.22	8.73	0.137	0.026
P22352	Glutathione peroxidase 3 (GPX3)	4.5	1	1	4.5	4.5	0.003	0.003
P04433	Ig kappa chain V-III region VG (Fragment)	11	5	5	2.2	2.2		0.027
P69905	Hemoglobin subunit alpha (HBA1)	182	56	74	3.25	2.46	0.042	0.027
P01743	Ig heavy chain V-I region HG3	9.5	4,5	7	2.11	1.36	0.019	0.038
P01767	Ig heavy chain V-III region BUT	10.5	6.5	14.5	1.62	−1.38	0.030	0.030
P01597	Ig kappa chain V-I region DEE	13.5	7.5	8	1.8	1.69	0.063	0.008
B9A064	Immunoglobulin lambda-like polypeptide 5	143.5	87	104.5	1.65	1.37	0.091	0.008
P02788	Lactotransferrin (LTF)	15	1	3.5	15	4.29	<0.001	0.009
P61626	Lysozyme C (LYZ)	13	8.5	6.5	1.53	2	0.061	0.04
P07737	Profilin-1 (PFN1)	29	15	22	1.93	1.32	0.025	0.02
P98160	Basement membrane-specific heparan sulfate proteoglycan core protein (HSPG2)	13	8	6	1.63	2.17	0.155	0.038
P04220	Ig mu heavy chain disease protein	61.5	39	16	1.58	3.84	0.118	0.03
P01620	Ig kappa chain V-III region SIE	36.5	23.5	27.5	1.55	1.33	0.143	0.006
P05109	Protein S100-A8 (S100A8)	13.5	6.5	7.5	2.08	1.8	0.047	0.014
P01871	Ig mu chain C region (IGHM)	85.5	56	23.5	1.53	3.64	0.176	0.048
P06702	Protein S100-A9 (S100A9)	17.5	10	11.5	1.75	1.52	0.022	0.014
P02766	Transthyretin (TTR)	126.5	77.5	101	1.63	1.25	0.004	0.005
O75112	LIM domain-binding protein 3 (LDB3)	1	9.5	29	−9.5	−29	0.02	<0.001
P05976	Myosin light chain 1/3, skeletal muscle isoform (MYL1)	1	19	19	−19	−19	0.007	0.001
Q96A32	Myosin regulatory light chain 2, skeletal muscle isoform (MYLPF)	1	23	25.5	−23	−25.5	<0.001	0.031
Q9UKX2	Myosin-2 (MYH2)	8.67	143	192	−16.5	−22.15	<0.001	0.03
P00441	Superoxide dismutase [Cu-Zn] (SOD1)	3.5	9	10	−2.57	−2.86	0.039	0.028

Peptide spectrum matches (PSMs) of each protein were used for quantitative analysis and only proteins with *p*-value ≤ 0.05 are shown. Proteins found significantly dysregulated also in DIA are highlighted.

**Table 4:** Summary of clinical and genetic data.

Pt ID	Dx	Age	Sex	EcoRI	CSS	Muscle	Age at Onset	Symptoms at Onset	Treatment	Past Medical History
p2	CTRL	24	M	N.P.	0	Vastus lateralis	N.A.	N.A.	None	Unremarkable
p4	FSHD	43	F	17	3	Gastrocnemius lateralis	Second decade	Scapular winging	Levothyroxine	Hypothyroidism
p5	FSHD	53	F	24	3.5	Peroneus	Second decade	Scapular winging	Levothyroxine	Hypothyroidism
p6	FSHD	55	F	15	3.5	Vastus lateralis	Second decade	Scapular winging	Amlodipine, bisoprolol, hydrochlorothiazide	Hypertension
p7	FSHD	29	M	20	2.5	Biceps femoris short head	Second decade	Difficulty in raising arms	None	Unremarkable
p8	FSHD	18	M	23	1.5	Semimembranosus	Second decade	Difficulty in raising arms	None	Unremarkable
p9	NPGC	47	F	33	0	Vastus lateralis	N.A.	N.A.	None	Headache
p10	FSHD	44	M	25	3.5	Gastrocnemius lateralis	Fourth decade	Scapular winging	None	Unremarkable
p11	FSHD	58	F	23	3	Gracilis	Second decade	Difficulty in raising arms	Cholecalciferol	Osteoporosis
p12	FSHD	53	M	24	3	Extensor digitorum longus	Fifth decade	Scapular winging	None	Unremarkable
p13	CTRL	60	M	>40	0	Vastus lateralis	N.A.	N.A.	None	Unremarkable
p14	CTRL	50	M	>40	0	Vastus lateralis	N.A.	N.A.	None	Unremarkable
p15	FSHD	34	M	19	3	Semitendinosus	Third decade	Facial weakness	None	Unremarkable
p17	FSHD	30	F	18	3	Tibialis anterior	Second decade	Scapular winging	None	Unremarkable
p18	CTRL	32	F	N.P.	0	Vastus lateralis	N.A.	N.A.	None	Unremarkable

Pt = patient, Dx = diagnosis, M = male, F = female, EcoRI = EcoRI fragment length in kb, N.P. = not performed, N.A. = not applicable, CSS = clinical severity scale, CTRL = control.

## Data Availability

The data that support the findings of this study are available from the corresponding author upon reasonable request.

## Supplementary Materials

Supplementary materials can be found at https://www.mdpi.com/1422-0067/22/1/290/s1. Supplementary methods, Tables S1–S9, Figures S1–S3. The mass spectrometry proteomics data have been deposited to the ProteomeXchange Consortium via the PRIDE [43] partner repository with the dataset identifier PXD021838.

## Abbreviations

FSHD	Facioscapulohumeral muscular dystrophy
MRI	Magnetic Resonance Imaging
STIR	Short Tau Inversion Recovery
LC-MS	Liquid Chromatography-Mass Spectrometry
NPGC	Non-penetrant gene carrier
DIA	Data Independent Acquisition
DDA	Data Dependent Acquisition
GO	Gene Ontology
PLGS	ProteinLynx Global Server
CTRL	Control
PSMs	Peptide Spectrum Matches
CSS	Clinical severity scale
FASP	Filter-aided sample preparation
MRM	Multiple reaction monitoring
IPA	Ingenuity Pathway Analysis
DAMP	Damage-Associated Molecular Pattern
MWCO	Molecular weight cut-off
