# Supplementary material for: Proteomics of Muscle Microdialysates Identifies Potential Circulating Biomarkers in Facioscapulohumeral Muscular Dystrophy
Source: Int J Mol Sci. 2020 Dec 30;22(1):290. doi: 10.3390/ijms22010290 (PMC7795508; doi:10.3390/ijms22010290)
Supplement: Supplementary file 1 [file ijms-22-00290-s001.zip › Supplementary material_rev/Table S5.docx]

**IPA (STIR- vs STIR+)**

**Experiment Metadata**

**Top Canonical Pathways**

Name p-value Overlap

**Clathrin-mediated Endocytosis Signaling** 5.88E-18 6.8 % 14/207

**Mechanisms of Viral Exit from Host Cells** 2.84E-10 14.6 % 6/41

**Remodeling of Epithelial Adherens**

**Junctions** 5.52E-09 9.1 % 6/66

**LXR/RXR Activation** 6.50E-09 5.8 % 7/121

**RhoA Signaling** 6.50E-09 5.8 % 7/121

**Top Upstream Regulators**

**Top Upstream Regulators**

**Upstream Regulators**

Name p-value Predicted Activation

**MRTFA** 2.62E-09 Activated

**SRF** 4.25E-09 Activated

**BCL11A** 1.03E-07

**MRTFB** 2.58E-07 Activated

**HOXA10** 5.49E-07

**Causal Network**

Name p-value Predicted Activation

**MRTFA** 1.26E-10 Activated

**SRF** 1.74E-10 Activated

**JINK1/2** 5.18E-10 Activated

**APOC2** 7.21E-10

**SETDB1** 3.36E-09

**Top Diseases and Bio Functions**

**Top Diseases and Bio Functions**

**Diseases and Disorders**

Name p-value range # Molecules

**Inflammatory Response** 1.68E-02 - 1.81E-10 29

**Metabolic Disease** 1.70E-02 - 1.95E-09 18

**Organismal Injury and Abnormalities** 1.71E-02 - 1.95E-09 35

**Neurological Disease** 1.63E-02 - 3.20E-08 22

**Psychological Disorders** 1.28E-02 - 3.20E-08 15

**Molecular and Cellular Functions**

Name p-value range # Molecules

**Protein Synthesis** 6.54E-03 - 9.39E-13 17

**Cellular Function and Maintenance** 1.70E-02 - 1.86E-11 18

**Cellular Movement** 1.70E-02 - 1.27E-08 22

**DNA Replication. Recombination. and Repair** 1.07E-02 - 3.08E-07 5

**Cellular Compromise** 6.41E-03 - 7.73E-07 16

**Physiological System Development and Function**

Name p-value range # Molecules

**Humoral Immune Response** 1.07E-02 - 1.81E-10 10

**Immune Cell Trafficking** 1.49E-02 - 1.27E-08 18

**Hematological System Develop. and Func.** 1.49E-02 - 3.32E-06 17

**Organismal Functions** 4.28E-03 - 1.17E-05 9

**Tissue Development** 1.40E-02 - 3.78E-05 16

**Top Networks**

ID Associated Network Functions Score

**1**

Metabolic Disease. 48

Organismal Injury and

Abnormalities. Protein

Synthesis

**2**

DNA Replication. 25

Recombination. and

Repair. Infectious

Diseases. Cell

Signaling

**3**

Cellular Movement. 13

Connective Tissue

Development and

Function. Cell

Morphology

**4**

Cell Morphology. 2

Cellular Assembly and

Organization. Cellular

Compromise

**5**

Humoral Immune 2

Response.

Inflammatory

Response. DNA

Replication.

Recombination. and

Repair

**Top T**

**Top Toxic Lists**

Name p-value Overlap

**LXR/RXR Activation** 7.29E-09 5.7 % 7/123

**NRF2-mediated Oxidative Stress Response** 5.03E-07 3.1 % 7/228

**FXR/RXR Activation** 6.85E-06 4.0 % 5/125

**Positive Acute Phase Response Proteins** 1.87E-03 6.7 % 2/30

**Negative Acute Phase Response Proteins** 1.70E-02 12.5 % 1/8

**Top My Lists**

**IPA (STIR+ vs CTRL)**

**Top Canonical Pathways**

Name p-value Overlap

**Clathrin-mediated Endocytosis Signaling** 2.30E-21 9.2 % 19/207

**LXR/RXR Activation** 9.42E-16 10.7 % 13/121

**Actin Cytoskeleton Signaling** 7.95E-15 6.6 % 15/228

**Regulation of Actin-based Motility by Rho** 3.15E-14 12.5 % 11/88

**RhoA Signaling** 3.45E-14 9.9 % 12/121

**Top Upstream Regulators**

**Upstream Regulators**

Name p-value Predicted Activation

**SRF** 1.43E-10

**NOS2** 6.72E-10

**MYOD1** 1.22E-09

**IL6** 4.91E-08

**AKT1** 5.59E-08

**Causal Network**

Name p-value Predicted Activation

**AVP** 4.72E-16

**SETDB1** 5.92E-14

**EDNRB** 1.03E-13

**MLK1/2/3** 1.07E-13

**NKX2-5** 1.71E-13

**Top Diseases and Bio Functions**

**Diseases and Disorders**

Name p-value range # Molecules

**[Inflammatory Response](https://analysis.ingenuity.com/pa/api/v2/analysissummary?applicationname=IPA_PDF_EXPORT&analysisuid=17779625)** [2.77E-03 - 1.86E-15 51](https://analysis.ingenuity.com/pa/api/v2/analysissummary?applicationname=IPA_PDF_EXPORT&analysisuid=17779625)

**Cardiovascular Disease** 2.63E-03 - 1.21E-12 37

**Organismal Injury and Abnormalities** 2.84E-03 - 1.21E-12 72

**Neurological Disease** 2.78E-03 - 2.01E-12 44

**Psychological Disorders** 1.90E-03 - 2.01E-12 34

**Molecular and Cellular Functions**

Name p-value range # Molecules

**Cellular Compromise** 1.17E-03 - 1.86E-15 27

**Protein Synthesis** 5.11E-04 - 1.15E-13 22

**Cellular Assembly and Organization** 2.63E-03 - 1.13E-12 28

**Cellular Function and Maintenance** 2.63E-03 - 4.17E-12 35

**Cellular Movement** 2.78E-03 - 1.34E-09 36

**Physiological System Development and Function**

Name p-value range # Molecules

**Humoral Immune Response** 4.00E-04 - 3.46E-10 9

**Immune Cell Trafficking** 2.77E-03 - 1.34E-09 27

**Skeletal and Muscular System Development and Function** 2.78E-03 - 2.13E-09 37

**Organismal Development** 2.63E-03 - 3.51E-09 43

**[Hematological System Development and Function](https://analysis.ingenuity.com/pa/api/v2/analysissummary?applicationname=IPA_PDF_EXPORT&analysisuid=17779625)** [2.78E-03 - 2.45E-07 33](https://analysis.ingenuity.com/pa/api/v2/analysissummary?applicationname=IPA_PDF_EXPORT&analysisuid=17779625)

**Liver Necrosis/Cell Death** 1.29E-01 - 2.13E-03 4

**Liver Cirrhosis** 5.74E-02 - 3.83E-03 3

**Nephrotoxicity**

Name p-value range # Molecules

**Nephrosis** 4.87E-02 - 1.50E-05 6

**Renal Damage** 1.19E-01 - 2.79E-04 8

**Glomerular Injury** 1.91E-01 - 3.48E-03 5

**Renal Tubule Injury** 5.60E-02 - 3.99E-03 4

**Kidney Failure** 1.91E-01 - 2.65E-02 3

**Top Regulator Effect Networks**

ID Regulators Disease & Functions Consistency Score DNMT3A.FOS.MEF2C.PPARG Accumulation of macrophages binding of neutrophils 4.221

**IPA (STIR- vs CTRL)**

**Top Canonical Pathways**

Name p-value Overlap

**LXR/RXR Activation** 6.42E-20 14.9 % 18/121

**Cellular Effects of Sildenafil (Viagra)** 1.37E-19 14.3 % 18/126

**Actin Cytoskeleton Signaling** 1.19E-18 9.2 % 21/228

**Epithelial Adherens Junction Signaling** 3.45E-18 12.0 % 18/150

**Calcium Signaling** 2.72E-17 9.6 % 19/198

**Top Upstream Regulators**

**Upstream Regulators**

Name p-value Predicted Activation

**DMD** 1.08E-18

**SRF** 5.68E-16 Inhibited

**IL6** 2.54E-13

**MYOD1** 3.93E-13

**OSM** 1.48E-11

**Causal Network**

Name p-value Predicted Activation

**OSM** 5.74E-21

**NKX2-5** 6.77E-21

**SETDB1** 1.44E-19

**ACOX1** 1.83E-19

**SSRP1** 2.13E-19

**Top Diseases and Bio Functions**

**Diseases and Disorders**

Name p-value range # Molecules

**[Inflammatory Response](https://analysis.ingenuity.com/pa/api/v2/analysissummary?applicationname=IPA_PDF_EXPORT&analysisuid=17779675)** [1.20E-03 - 6.92E-24 79](https://analysis.ingenuity.com/pa/api/v2/analysissummary?applicationname=IPA_PDF_EXPORT&analysisuid=17779675)

**Cancer** 1.20E-03 - 1.78E-17 118

**Dermatological Diseases and Conditions** 9.05E-04 - 1.78E-17 45

**Organismal Injury and Abnormalities** 1.20E-03 - 1.78E-17 120

**Metabolic Disease** 1.10E-03 - 4.56E-16 49

**Molecular and Cellular Functions**

Name p-value range # Molecules

**Cellular Assembly and Organization** 9.04E-04 - 4.13E-24 40

**Cellular Compromise** 1.20E-04 - 6.92E-24 48

**Cellular Movement** 1.18E-03 - 7.49E-15 58

**Cellular Function and Maintenance** 8.28E-04 - 8.63E-13 60

**Protein Synthesis** 1.61E-04 - 1.45E-10 32

**Physiological System Development and Function**

Name p-value range # Molecules

**Skeletal and Muscular System Development and Function** 1.07E-03 - 1.08E-20 45

**Immune Cell Trafficking** 1.16E-03 - 7.49E-15 48

**Humoral Immune Response** 1.10E-03 - 1.03E-10 14

**Organismal Development** 1.07E-03 - 4.62E-10 64

**[Hematological System Development and Function](https://analysis.ingenuity.com/pa/api/v2/analysissummary?applicationname=IPA_PDF_EXPORT&analysisuid=17779675)** [1.16E-03 - 3.55E-09 55](https://analysis.ingenuity.com/pa/api/v2/analysissummary?applicationname=IPA_PDF_EXPORT&analysisuid=17779675)

**Top Regulator Effect Networks**

ID Regulators Disease & Functions Consistency Score

**1** EGR1 Cell survival 1.5

**2** PPARG Transport of lipid 0.894

**3** TGFB1 Necrosis of tumor -12.965

**4** TGFB1 Adhesion of epithelial cells -19.677

**IPA (STIR+ vs STIR- vs CTRL vs NPGC)**

**Top Canonical Pathways**

Name p-value Overlap

**LXR/RXR Activation** 2.29E-21 14.9 % 18/121

**Acute Phase Response Signaling** 7.93E-20 10.9 % 19/175

**FXR/RXR Activation** 1.56E-19 13.6 % 17/125

**Epithelial Adherens Junction Signaling** 1.78E-15 10.3 % 15/146

**Complement System** 2.10E-13 25.0 % 9/36

**Top Upstream Regulators**

**Upstream Regulators**

Name p-value Predicted Activation

**PSEN1** 3.59E-11

**TGFB1** 4.41E-09

**MAPT** 5.10E-09

**CEBPB** 1.37E-08

**IL6** 2.75E-08

**Causal Network**

Name p-value Predicted Activation

**RHOD** 2.08E-15

**DSP** 1.19E-14

**AVP** 3.69E-14

**Mlc** 7.45E-14

**RND3** 9.36E-14

**Top Diseases and Bio Functions**

**Diseases and Disorders**

Name p-value range # Molecules

**Inflammatory Response** 1.14E-04 - 1.36E-20 68

**Neurological Disease** 1.14E-04 - 8.00E-17 47

**Organismal Injury and Abnormalities** 1.35E-04 - 8.00E-17 83

**Psychological Disorders** 6.50E-05 - 8.00E-17 44

**Metabolic Disease** 2.83E-05 - 1.24E-16 42

**Molecular and Cellular Functions**

Name p-value range # Molecules

**Cellular Compromise** 1.41E-10 - 8.39E-15 26

**Protein Synthesis** 9.65E-06 - 6.39E-13 28

**Cellular Function and Maintenance** 1.17E-04 - 7.77E-12 26

**Cellular Movement** 9.34E-05 - 1.22E-10 47

**Cell Death and Survival** 1.14E-04 - 1.63E-10 18

**Physiological System Development and Function**

Name p-value range # Molecules

**Humoral Immune Response** 8.14E-06 - 1.36E-20 17

**Immune Cell Trafficking** 9.34E-05 - 6.28E-10 36

**Skeletal and Musc. System Develop. and Func.** 4.08E-05 - 9.17E-08 12

**Hematological System Develop. and Func.** 1.34E-04 - 1.23E-07 30

**Tissue Development** 1.17E-04 - 6.70E-06 17
