# Supplementary material for: Proteomics of Muscle Microdialysates Identifies Potential Circulating Biomarkers in Facioscapulohumeral Muscular Dystrophy
Source: Int J Mol Sci. 2020 Dec 30;22(1):290. doi: 10.3390/ijms22010290 (PMC7795508; doi:10.3390/ijms22010290)
Supplement: Supplementary file 1 [file ijms-22-00290-s001.zip › Supplementary material_rev/Supporting Information.docx]

**Proteomics of muscle microdialysates identifies potential circulating biomarkers in Facioscapulohumeral muscular dystrophy**

Victor Corasolla Carregari^1^, Mauro Monforte^2^, Giuseppe Di Maio^1^, Luisa Pieroni^3^, Andrea Urbani^1^, Enzo Ricci^2,4^, Giorgio Tasca^2^

^1^ Istituto di Biochimica e Biochimica Clinica, Università Cattolica del Sacro Cuore, Roma, Italia.

^2^ Unità Operativa Complessa di Neurologia, Fondazione Policlinico Universitario A. Gemelli IRCCS, Roma, Italia

^3^ Unità di Proteomica e Metabolomica, Fondazione S. Lucia IRCCS, Roma, Italia

^4^ Istituto di Neurologia, Università Cattolica del Sacro Cuore, Roma, Italia.

**Supporting Information**

**Table S1.** Proteins identified by the Protein Lynks Global Server in DIA mode in the microdialysate samples from STIR+, STIR- and control muscles.

**Table S2.** List of all the proteins identified in DDA mode in the microdialysate samples from STIR+, STIR- and control muscles and differentially regulated proteins in STIR + compared with STIR- and control samples. Proteome Discoverer 2.2 was used as software engineer and only proteins with a p.value < 0.05 were considered. Label Free analysis was performed using the PSM (Peptide Spectra Matches) count approach.

**Table S3.** Proteins overlapped in all the conditions (STIR+, STIR-, CTRL and NPGC) respecting the quantitative parameters.

**Table S4.** Upstream gene regulators based on the fold-change level of protein dysregulation in all tested conditions.

**Table S5.** Ingenuity Pathway Analysis of proteins regulated in all tested conditions.

**Table S6.** Comparison of protein expression between the STIR+ and control samples. The differences between the protein levels are shown as ratio. Only proteins with more than 20% fold change are listed.

**Table S7.** Comparison of protein expression between the STIR- and control samples.

**Table S8**. Detailed list of all proteins identified in the serum samples.

**Table S9.** Comparison of protein expression between the sera of FSHD and healthy control patients.

**Figure S1.** Paired comparison between STIR+ and STIR- samples from the same patient using the Wilcoxon signed rank test applying Benjamini-Hochberg FDR correction for multiple comparisons. On y axis the log2 of the relative abundance of the peak intensity of all peptides belonging to the mentioned protein is shown. Fold-change cut-off for dysregulation was set to ±20% and a p-value < 0.05 was considered statistically significant.

**Figure S2.** A) IPA Network analysis of regulated proteins in STIR+ compared with STIR- samples. B) IPA Network analysis of regulated proteins in STIR+ compared with control samples. C) IPA Network analysis of regulated proteins in STIR- compared with control samples.

**Figure S3.** A) Overlap between proteins identified in FSHD serum and STIR+ microdialysates. C) Overlap between proteins found only in sera from FSHD patients (and absent in controls) and those dysregulated in STIR+ vs STIR- microdialysates.
