# Supplementary figures and images for: Proteomics of Muscle Microdialysates Identifies Potential Circulating Biomarkers in Facioscapulohumeral Muscular Dystrophy
Source: Int J Mol Sci. 2020 Dec 30;22(1):290. doi: 10.3390/ijms22010290 (PMC7795508; doi:10.3390/ijms22010290)

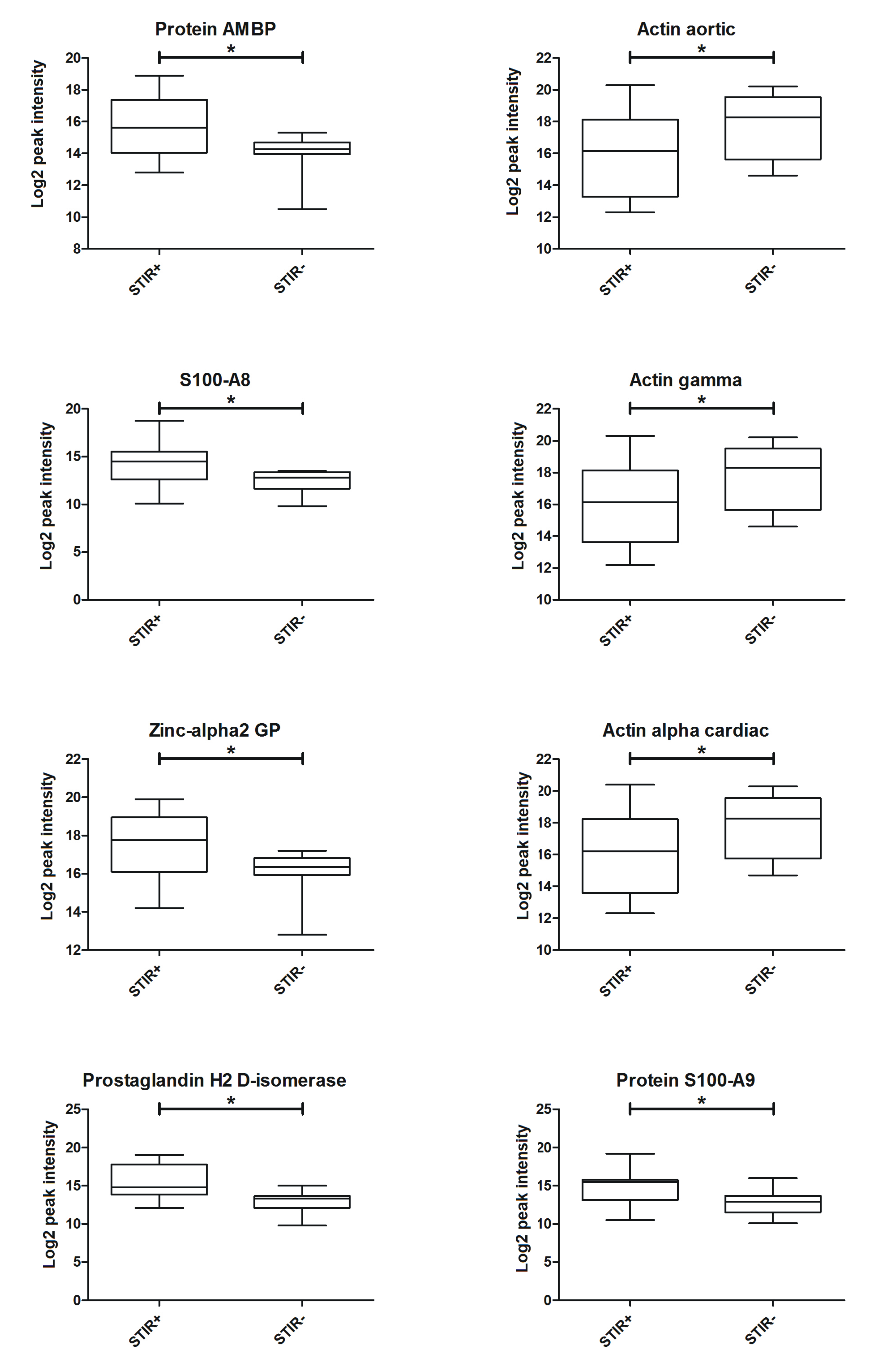

Supplement: Supplementary file 1 [file ijms-22-00290-s001.zip › Supplementary material_rev/Figure S1.TIF]

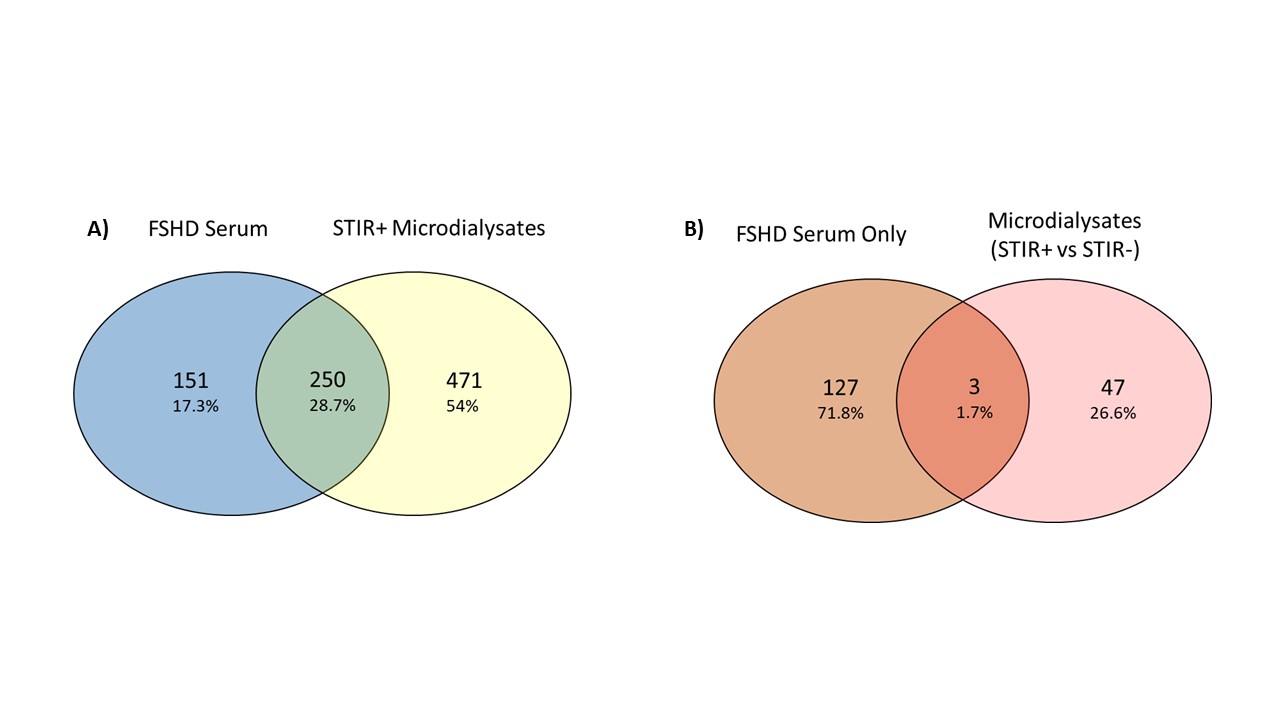

Supplement: Supplementary file 1 [file ijms-22-00290-s001.zip › Supplementary material_rev/Figure S3.jpg]

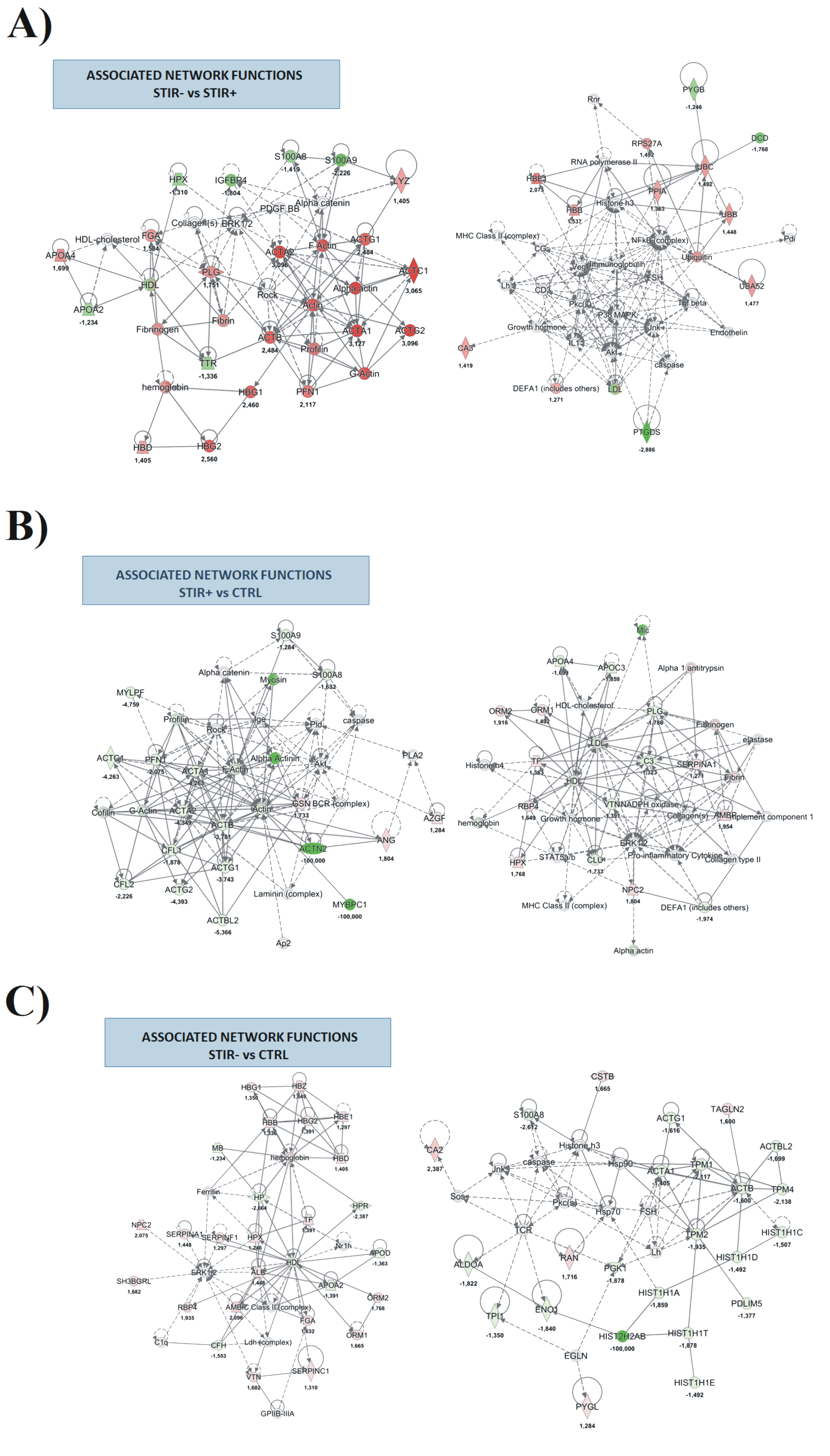

Supplement: Supplementary file 1 [file ijms-22-00290-s001.zip › Supplementary material_rev/Figure S2.jpg]
