# Supplementary material for: Proteomics of Muscle Microdialysates Identifies Potential Circulating Biomarkers in Facioscapulohumeral Muscular Dystrophy
Source: Int J Mol Sci. 2020 Dec 30;22(1):290. doi: 10.3390/ijms22010290 (PMC7795508; doi:10.3390/ijms22010290)
Supplement: Supplementary file 1 [file ijms-22-00290-s001.zip › Supplementary material_rev/Supplementary Methods.doc]

**Proteomics of muscle microdialysates identifies potential circulating biomarkers in Facioscapulohumeral muscular dystrophy**

Victor Corasolla Carregari^1^, Mauro Monforte^2^, Giuseppe Di Maio^1^, Luisa Pieroni^3^, Andrea Urbani^1^, Enzo Ricci^2,4^, Giorgio Tasca^2^

^1^ Istituto di Biochimica e Biochimica Clinica, Università Cattolica del Sacro Cuore, Roma, Italia.

^2^ Unità Operativa Complessa di Neurologia, Fondazione Policlinico Universitario A. Gemelli IRCCS, Roma, Italia

^3^ Unità di Proteomica e Metabolomica, Fondazione S. Lucia IRCCS, Roma, Italia

^4^ Istituto di Neurologia, Università Cattolica del Sacro Cuore, Roma, Italia.

**Supporting Methods**

**Synapt G2si (DIA): setting of the instrument, label free quantification and statistical analysis**

The mass spectrometer operated in HDMSE mode, a DIA acquisition protocol, switching between low (4 eV) and high (15–40 eV) collision energies on the gas cell, using a scan time of 1.5 s per function over 50–2000 m/z. The processing through low and elevated energy, added to the data of the reference lock mass ([Glu1]-Fibrinopeptide B Standard, Waters Corp.), provides a time-aligned inventory of accurate mass-retention time components for both low and elevated-energy (EMRT, exact mass retention time). The qualitative identification of proteins was obtained by searching in the UniProt KB database release of June 2018, restricted respectively to Homo Sapiens, to which the sequence of PHS2_RABIT GLYCOGEN PHOSPHORYLASE (UniProtKB/Swiss-Prot AC: P00489) was appended. Search parameters were set as: automatic tolerance for precursor ions and for product ions, minimum 1 fragment ions matched per peptide, minimum 3 fragment ions matched per protein, minimum 2 peptide matched per protein, 1 missed cleavage, carbamidomethylation of cysteines as fixed modification and oxidation of methionines as variable modifications, false discovery rate (FDR) of the identification algorithm < 1%, and 500 fmol as the concentration of the reference standard.

Label free quantitative analysis was obtained with the Protein Expression Analysis Mode integrated in PLGS software, using the internal standard for normalization. The expression analysis was performed considering technical replicates available for each experimental condition (i.e, STIR+, STIR- and CTRL) following the hypothesis that each group is an independent variable. Filtered tables were generated to include proteins found in at least two out of three technical replicates and to exclude proteins showing less than 20% change and those showing no statistical significance according to the PLGS software. A value of P ranging between 0.95-1.00 indicates that the cluster is upregulated; a value of P between 0.05-0.00 indicates that the cluster is downregulated.

For label free comparison between STIR+ and STIR- samples we only considered proteins that were present in 8 of 10 samples for each condition. A paired comparison between STIR+ and STIR- samples from the same patient was performed to reduce variance; Wilcoxon signed rank test applying Benjamini-Hochberg FDR correction for multiple comparisons was used. Fold change cut-off for dysregulation was set to ±20% and a p-value < 0.05 was considered statistically significant.

**Obitrap Elite (DDA): setting of the instrument, label free quantification and statistical analysis**

One volume of sample was mixed with ten volumes of Isopropanol (IPA)-1% TCA solution. The sample was then vigorously vortexed for two minutes and then centrifuged at 1500 g for 5 min at 5°C. The first supernatant (F1) was then collected and stored at -80°C for further processing. The pellet was re-suspended in methanol and then centrifuged at 2000 rpm and the supernatant (F2) was stored at -80°C for further processing. The final protein pellet (F3) was re-suspended in 30 µl of 100mM ammonium bicarbonate (AmBic). F1 and F2 were then dried by lyophilization and resuspended in 30 µl of AmBic 100mM.

For all the fractions, a Cysteine reduction and alkylation was performed through the addition of 1:10 (v/v) DTT 100mM followed by incubation for 1h at 37°C in thermomixer under agitation and then of 1:10 (v/v) Iodoacetamide 200mM, followed by another incubation for 1h at 37°C under agitation. DTT 100mM 1:62 (v/v) was added to quench the free Iodoacetamide. After reduction and alkylation Trypsin was added with a ratio of 1:50 of the total protein amount. The samples were then incubated for 16 hours at 37°C and the reaction was stopped by adding 1 µl of Formic Acid 10%. To desalt the tryptic peptides obtained from digestion, Zip-Tip C18 was performed according to the manufacturer's instructions with elution steps at 50% and 80% Acetonitrile (ACN). The eluted peptides were then lyophilized to remove ACN from the samples and finally resuspended in 13µl of 0.1% (v/v) Formic Acid (FA) aqueous solution ready for MS analysis.

HPLC-ESI-MS/MS experiments were performed on a Dionex UltiMate 3000 RSLC nanoSystem (Thermo Fisher Scientific, Waltham, MA, USA) directly coupled to an Orbitrap Elite Hybrid Ion Trap-Orbitrap mass spectrometer (Thermo Fisher Scientific) with nanoESI source (EASY-Spray). The injection volume was set to 5 μl and peptides were first trapped on a Acclaim PepMap100 C18 µ-Precolumn (5 µm particle size, 100 Å pore size, 300 µm i.d. x 5 mm, Thermo Fisher Scientific) for 5 min at 10 μl/min in an aqueous solution of 0.1% FA and then separated by reverse-phase chromatography performed on Acclaim PepMap RSLC C18 column (2 μm particle size, 100 Å pore size, 50 μm i.d. x 150 mm, Temperature 40°C, Thermo Fisher Scientific) at a flow rate of 300 nl/min, using an aqueous solution of 0.1% FA as eluent A and ACN with 0.1 % FA as eluent B. The following chromatographic conditions were used: linear gradient from 5 to 35% B in 120 min and total LC-run of 160 min. Mass spectra were collected by the mass spectrometer operating in positive ion mode and data dependent scan mode in which each full mass spectrometric scan (mass range 350-2000 m/z, mass analyzer Orbitrap, resolution of 60000) was followed by data dependent fragmentation experiments (MS/MS) of the twenty most abundant multiple-charged precursor ions via collision-induced dissociation (CID, mass analyzer Ion Trap). Protein identifications were obtained with the embedded ion accounting algorithm (Sequest HT) of the software Proteome Discoverer (version 1.4, Thermo Fisher Scientific) after searching a UniProtKB/Swiss-Prot Protein Knowledgebase (Release 2015_06 of 27-May-15; taxonomical restriction Homo sapiens). The search parameters were 5 ppm tolerance for precursor ions and 0.5 Da for product ions, 1 missed cleavage, carbamydomethylation (+57.021 Da) of cysteine as fixed modification, oxidation (+15.995 Da) of methionine as variable modification, minimum protein score of 5, and false discovery rate under 0.01 (i.e.: the expected fraction of incorrect peptide spectrum match in the entire data set is less than 1%, calculated on a decoy database).

In order to evaluate the relative abundance of the same protein in different samples, spectral counts label free quantitative method has been used on fraction merged data. With this method MS/MS spectra, matched to a given protein database, allow the determination of relative abundance by comparing the number of identified MS/MS spectra from the same protein in each of the multiple LC-MS/MS datasets. Significance of the differences of average spectral counts in the different conditions (STIR+; STIR-; CTRL) was analyzed with multiple Student's t-test. P values <0.05 were considered significant and analyzed without assuming a consistent SD.

**Ingenuity Pathway Analysis**

The analysis parameters were set as: Direct and indirect relationship; endogenous chemical substances included; all molecules and/or relationships considered as Summary Filter. The most significant categories associated with the loaded datasets were identified by calculating their significance (p-value, Fischer test). A p-value threshold of 0.05 has been set as significant for an association between gene/proteins present in the data sets and each pathway (Canonical pathway, Upstream regulators and Biological Function).

**Multiple Reaction Monitoring (MRM)**

Digested peptides from STIR+, STIR- and control samples were pooled and a total of 5 DUX4 proteins targets (ZSCAN4, PRAMEF1, PRAMEF2, DEFB103, and MBD3L2) and DUX4 itself were chosen and submitted to theorical trypsin digestion, isolating the mass of peptides of interest, used for the MS/MS selection in the MRM method. Briefly, the fasta sequence of the proteins of interest were theoretically digested by trypsin allowing 1 miss cleavage (PeptideMass; ExPASy bioinformatic resource portal) and the monoisotopic mass of selected fragments (peptides containing more than 8 and less than 25 amino acids) were inserted in Target Lynx a MassLynx software (Waters Corp.) and performed an MRM- run choosing the mass/charge (m/z) range of precursor ions from 50 to 1500 Th (Thompson units) and 0.055 Th for mass tolerance.

**Protein ontologies and network analysis**

To identify biologically relevant molecular pathways the proteomic dataset was analyzed by a bioinformatic analysis tool based on QIAGEN’S Ingenuity Pathways Analysis (QIAGEN; Inc., https://www.qiagenbioinformatics.com/products/ingenuity-pathway-analysis). For the Gene Ontology analysis we made use of the PANTHER 14.0 (Protein Analysis THrough Evolutionary Relationships) Classification System (GENEONTOLOGY Unifying Biology) containing 15.552 protein families, and STRING: functional protein association networks, v.11.0.

**Heatmap**

The heatmap was built using *pheatmap* package of the R software ([https://www.r-project.org](https://www.r-project.org/)). The median value of the 3 normalized log2 technical replicates of the sum of the Intensity Peaks for each protein identified in each patient was used to calculate a Z-score of protein abundance. Only proteins identified in 8 out of 10 STIR+ and STIR- samples and in all the control samples were considered. Average Linkage method and the Pearson correlation were used to calculate the clustering distance between samples.
